# Neuroimaging in pediatric language development and disorders: a scoping review protocol

**DOI:** 10.1186/s13643-025-02969-y

**Published:** 2025-11-21

**Authors:** Ruochen Ning, Karla N. Washington

**Affiliations:** Department of Speech-Language Pathology, University of Toronto, 500 University Avenue, Toronto, Canada

**Keywords:** Neuroimaging, Language development, Language disorders, Language acquisition, Language network

## Abstract

**Background:**

Infancy and early childhood represent critical periods for language development, as well as for the diagnosis and intervention of language disorders. Neuroimaging techniques, such as functional magnetic resonance imaging (fMRI), diffusion tensor imaging (DTI) and magnetoencephalography (MEG), have revolutionized our understanding of the brain’s role in language development and disorders. These techniques provide detailed insights into the neural mechanisms underlying language acquisition and the deviations associated with language disorders. However, there is a notable lack of comprehensive literature reviews on the application of neuroimaging techniques in studying pediatric language development and disorders, particularly in children under eight years old in the field of speech-language pathology. This gap in the literature hinders the ability to form a cohesive understanding of the current state of research and its clinical implications, i.e., garnering an understanding of the contributions of these technological tools in understanding language development and disorders.

**Methods:**

To address this gap, we will conduct a scoping review to synthesize literature involving neuroimaging techniques that examines language development and disorders in typically developing children and those diagnosed with language disorders under the age of eight. This review will be guided by the methodological framework proposed by Arksey and O’Malley [18]. A comprehensive and systematic search will be performed across multiple databases (MEDLINE, Embase, EBSCO CINAHL, PsycINFO, SCOPUS, and The Cochrane Library) to identify relevant peer-reviewed publications as well as grey literature. Studies will be screened according to predefined inclusion criteria. Key data from eligible studies will be extracted, synthesized and presented using both quantitative (numerical) and qualitative (narrative) approaches to address the research questions.

**Discussion:**

This scoping review aims to provide a comprehensive overview and summary of the methodologies and key findings in neuroimaging studies related to pediatric language development and disorders. The results will identify critical gaps in the current research, highlight the strengths and limitations of various neuroimaging techniques, and suggest future research directions in the field of pediatric neuroimaging for language development.

**Systematic review registration:**

Open Science Framework (osf.io/5jhk6).

**Supplementary Information:**

The online version contains supplementary material available at 10.1186/s13643-025-02969-y.

## Background

Language development is a critical aspect of early childhood, laying the foundation for cognitive, social, and academic success [1, 2]. Abnormal language development, usually referred to as language disorders, however, can emerge at various stages and across different domains of language development, potentially resulting in limited vocabulary, reading difficulties, and challenges in using language effectively for everyday communication [3]. Such impairments may have detrimental effects on children’s socioemotional well-being, cognitive development, and academic achievement [4]. Studying language development and disorders, especially among pediatric populations, is crucial for understanding this foundation and identifying early signs of atypical trajectories. Understanding language disorders at an early stage can also mitigate long-term effects on academic achievement and social integration, contributing to more effective therapeutic strategies and better quality of life outcomes for affected individuals. With the advancement of technologies, neuroimaging techniques, such as functional magnetic resonance imaging (fMRI), Positron Emission Tomography (PET), and magnetoencephalography (MEG), have revolutionized our ability to study the brain’s role in language development. These techniques are having increasing presence in diagnosing language-related disorders and in studying the underpinning neural correlates. Thus, it is important to know what neuroimaging techniques have been used in language development and disorder research and how they contribute to our understanding of this domain.

Following an extensive literature search, the only scoping review explicitly addressing the intersection of neuroimaging and pediatric language development was conducted by Costa et al., [5]. This review synthesized neuroimaging evidence from studies involving children engaged in writing tasks, a highly specific aspect of language development. Additionally, an overview by Abbot and Love [6] and six systematic reviews [7–12] have focused on specific questions on language-related brain structures and neural networks. However, the existing literature lacks a comprehensive overview of the application of neuroimaging techniques in pediatric language development and disorders. Furthermore, it does not adequately address the contributions and challenges associated with these advanced techniques.

This scoping review aims to fill in this gap by systematically mapping the existing literature on the use of neuroimaging techniques in studying language development and language disorders in preschool and early school age children. Neuroimaging research on language skills has predominantly focused on children aged six and older, as well as adults, due to the complexity of task requirements and scanning procedures [13, 14]. However, infancy (0–2 years old) and early childhood (2–8 years old), are critical periods for language development, which has been understudied in this context. During this period, children’s brains develop foundational skills that support future communication, literacy, and cognitive abilities [15]. Thus, a scoping review can help identify existing studies involving this population and highlight areas where further research is needed. By focusing on both typically developing children and those with language disorders, this review seeks to identify key findings, methodological approaches, and gaps in the current research domain. A scoping review was selected over a systematic review, as it allows for the examination of a wide breadth of sources related to exploratory questions, rather than seeking to answer a narrowly defined research question [16, 17]. The objective of this review is to investigate the neuroimaging techniques employed in studies involving young children, assess the contributions of neuroimaging to research on pediatric language development, and identify the associated challenges and limitations. Given the breadth and exploratory nature of these questions, a scoping review provides an appropriate methodological framework.

Through this review, we hope to provide a comprehensive overview of the current state of research in this field, highlight the contributions of neuroimaging to our understanding of pediatric language development, and identify areas where further research is needed. This will ultimately contribute to the development of more targeted and effective interventions for children with language disorders. The following research questions will be addressed:

1. What neuroimaging techniques have been utilized to study language development and language disorders in pediatric populations?
2. How does neuroimaging contribute to understanding the neural mechanisms underlying language development and language disorders in children under eight years old?
3. What are the challenges and limitations of using neuroimaging techniques in pediatric language development and disorder research?

## Methods

This scoping review follows the methodological framework outlined by Arksey and O’Malley [18], which provides a comprehensive approach to mapping the existing literature on a given topic. The framework involves six key stages: (1) identifying the research question; (2) identifying relevant studies; (3) study selection; (4) charting the data; (5) collating, summarizing, and reporting the results; and (6) consultation exercise. Additionally, we have incorporated recommendations from Levac, Colquhoun, and O’Brien [19] to enhance the rigor and transparency of our review process.

To ensure the quality and transparency of our review, we also adhered to the PRISMA-ScR (Preferred Reporting Items for Systematic Reviews and Meta-Analyses extension for Scoping Reviews, [20]) statement and checklist (see Appendix 1). The PRISMA guidelines provide a standardized approach to reporting systematic reviews and meta-analyses, which we have adapted the extension for scoping review to ensure comprehensive and transparent reporting of our methodology and findings. This scoping review protocol was registered with the Open Science Framework (OSF) on October 29, 2024, and is publicly accessible at osf.io/5jhk6.

### Stage 1: Identifying the research question

Scoping study research questions are broad in nature as the focus is on summarizing breadth of evidence. However, they must also be clearly defined to guide and inform subsequent stages of the research process [19]. In this review, we developed clear and focused research questions to ensure a structured approach:

1. What neuroimaging techniques have been utilized to study language development and language disorders in pediatric populations?
2. How does neuroimaging contribute to understanding the neural mechanisms underlying language development and language disorders in children under eight years old?
3. What are the challenges and limitations of using neuroimaging techniques in pediatric language development and disorder research?

### Stage 2: Identifying relevant studies

The search strategy was designed in collaboration with an experienced university librarian. In this process, the search strategy parameters, concept maps, and trial search queries were defined, and keywords and concepts were modified to maximize the effectiveness and sensitivity of the search according to each database’s unique features.

The search strategy involved using a combination of keywords and MeSH terms related to neuroimaging, language development, language disorders, and pediatric populations, and identified 5248 citations when executed in MEDLINE (see Appendix 2 for MEDLINE search strategies). The neuroimaging terms were retrieved from the relevant pediatric neuroimaging literature [21–23] and aforementioned systematic reviews in this field, include: magnetic resonance imaging (MRI), functional MRI (fMRI), voxel-based morphometry (VBM), MR Perfusion, MR spectroscopy, diffusion imaging (DI), diffusion tensor imaging (DTI), diffusion weighted imaging (DWI), magnetoencephalography (MEG), positron emission tomography (PET), single photon emission computed tomography (SPECT), functional near-infrared spectroscopy (fNIRS), computed tomography (CT), and ultrasound. The language development and disorder terms were established by two authors who are speech-language pathologist and linguist and have rich research experiences, include: language development, language functioning, language processing, language acquisition, language learning, language ability, language disability, language network, language delay, language disorders/impairments, developmental language disorders, specific language impairments, agraphia, anomia, dyslexia, grammar, semantics, syntax, vocabulary, lexicon, word, sentence, literacy, story-telling, narrative, reading, and writing.

We tested the search strategy using a set of 10 relevant studies. Initially, we reviewed the titles, abstracts, and keywords of five studies to identify any key terms potentially missing from the search strategy. After confirming that all pertinent terms were included, we conducted the search in MEDLINE to determine whether the remaining five studies could be retrieved. The results indicated that all five studies appeared in the search output, thus demonstrating the effectiveness of the search strategy.

The MEDLINE search strategy will be translated in OVID Embase, EBSCO CINAHL, OVID PsycINFO®, SCOPUS and The Cochrane Library. Reference lists of included studies will be searched for citations.

We will conduct a thorough search of the grey literature to identify any non-indexed literature, including dissertation abstracts, government documents, conference proceedings, educational materials and reports, through OpenGrey, Canadian Agency for Drugs and Technologies in Health (CADTH), Conference Proceedings Index, and ProQuest Dissertations and Theses.

Searches will be limited to literature reported in English due to the high costs that may be caused by translation work. There will not be date or study type restrictions. Bibliographic information of included literature will be amalgamated and stored using the review management program, Covidence, all the duplicates will be removed before the screening.

### Stage 3: Study selection

Studies will be selected based on predefined inclusion criteria. These criteria include the following – participants were: (1) children under eight years old; (2) typically developing children or children with language disorders; and studies should (3) involve neuroimaging techniques as research method; and (4) have a primary focus on language development and/or language disorders.

The exclusion criteria are: (1) children over eight years old, or adults, or mixed age groups and children under eight years old were not reported separately; (2) participants were diagnosed with other types of disorders such as autism spectrum disorder and attention-deficit/hyperactivity disorder; (3) the study did not use any neuroimaging techniques, or used several techniques and neuroimaging outcomes were not reported separately; and (4) the primary focus was not language development nor language disorders.

A screening form for the title and abstract screening and another for the full text screening have been developed (see Appendix 3 and 4) and will be pilot tested separately with 10 articles.

During the first round of screening, the titles and abstracts will be reviewed independently against the inclusion criteria by two reviewers both in Covidence (to make the process visible and transparent among all the team members) and in the screening form (to annotate clearly the reason of exclusion or being uncertain). Additional citation information, including authors’ names and journal names, will be withheld from reviewers to prevent potential bias. Studies will be marked as “Yes”, “No” or “Maybe”, and annotations should be made in the screening form for those marked as “No” or “Maybe” to explain the reason. All reviewers had experience in speech-language pathology and literature review. Discrepancies will be resolved by discussion and consensus between the two reviewers, and with a third reviewer in case no agreement can be reached.

During the second round of screening, full text articles will be retrieved for studies marked as “Yes” or “Maybe” and will be reviewed independently against the inclusion criteria by two reviewers. The studies will be marked as “Include” or “Exclude” in both Covidence and the screening form to guarantee a transparent work process among the team members and clear annotations of the reason of exclusion, as the title and abstract screening stage. After the screening, any difference in the final decision of inclusion will be discussed among team members until a consensus is reached.

We will include published and unpublished literature reporting any quantitative, qualitative, mixed or multimethod research, including both longitudinal and cross-sectional methods.

**Fig. 1:**
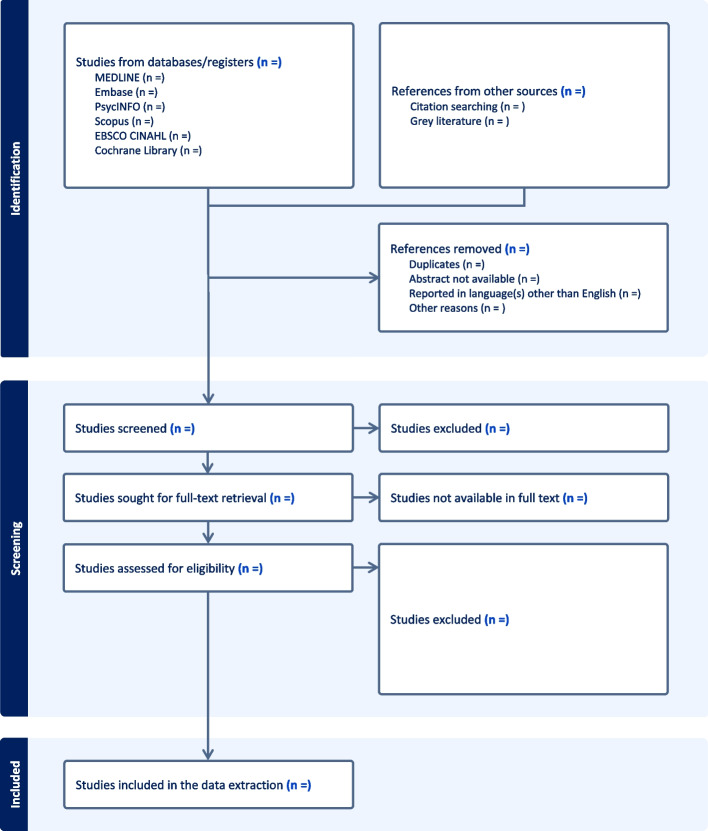
Process and results of the study selection

At the end of the study selection stage, the whole process will be presented with the following flow diagram (Fig. 1).

### Stage 4: Charting the data

All included studies will be reviewed and charted independently by two reviewers. A data extraction form (see Appendix 5) has been developed for this stage and will be pilot tested with 10 articles to ensure consistency. Necessary adjustments will be made and shared with all the team members prior to abstracting the remaining literature. The following data will be extracted when available (may vary based on included literature): authors, publication year, publication type (published and unpublished), study type (e.g., longitudinal, cross-sectional, report, commentary), study location, number of participants, age group and gender, participants’ condition (typically developing or diagnosed with language disorders), participants’ language background, neuroimaging techniques involved, primary focus (language development, language disorders), outcome measures, and outcomes/main findings.

### Stage 5: Synthesis and presentation of data

To provide a clear overview of all the information retrieved, and to establish the extent and nature of the literature, the results of the review will be presented using two strategies: (1) a basic numerical overview of the amount, type and distribution of included studies; and (2) a qualitative thematic analysis and mapping of the results.

We will create a table of included studies, listing for each: study types, aims, population, methods, outcome measures and outcomes, followed by a narrative synthesis of the included studies. We anticipate the charting and synthesis of the data will be an iterative process that depends on the literature included. Neither a study quality assessment nor a meta-analysis will be conducted as they are not part of the objective of the study or the scoping review methodology.

### Stage 6: Consultation with stakeholders

For this review, the primary stakeholders are clinical speech-language pathologists (SLPs) and neuroimaging practitioners. SLPs are represented within the research team, ensuring that clinical perspectives are integrated throughout the review process. Following the data synthesis and preliminary presentation of findings, we will conduct a targeted consultation with neuroimaging practitioners affiliated with hospitals and imaging centers that maintain established collaborative relationships with our laboratory. This consultation will focus on gathering expert feedback regarding the interpretation of findings, particularly in relation to methodological challenges and clinical applicability in pediatric neuroimaging. Insights from these practitioners will be systematically incorporated into the final interpretation and discussion sections of the review, with particular emphasis on addressing real-world barriers and enhancing the translational relevance of the results. This engagement will help ensure that the review is grounded in practical expertise and aligned with current clinical and research practices.

## Discussion

This paper presents the protocol for a scoping review of both peer-reviewed and grey literature on the use of neuroimaging techniques in studying language development and disorders of children under eight years old. This review will provide a comprehensive overview of current landscape, facilitate our knowledge on the neuroimaging technical advancement and application in pediatric language development research by clearly mapping the existing methodologies, key findings, and outcomes.

This review can provide a pivotal point in informing speech-language researchers and practitioners about existing and prior understandings regarding the application of neuroimaging techniques and interpreting their outcomes. Collectively, professionals in the speech-language pathology field can be better informed regarding the underpinnings of neural correlates of language development and language disorders in preschool and early school children. This synthesis will be essential for identifying gaps in literature, guiding future research directions, and fostering a deeper understanding of how different neuroimaging techniques contribute to our knowledge of pediatric language development and disorders.

For researchers, this review will serve as a valuable resource for designing robust studies and avoiding redundant efforts. For clinicians, it will offer insights into the most effective diagnostic and therapeutic approaches, ultimately improving the care and outcomes for young children with language disorders. By bringing together diverse studies, this scoping review will bridge the gap between research and clinical practice, promoting evidence-based interventions and enhancing the overall quality of pediatric language disorder management.

## Supplementary Information

Supplementary Material 1. Appendix 1: PRISMA-ScR checklist

Supplementary Material 2. Appendix 2: Search strategy for Medline

Supplementary Material 3. Appendix 3: Title and abstract screening form

Supplementary Material 4. Appendix 4: Full text screening form

Supplementary Material 5. Appendix 5: Data extraction form

## Data Availability

Data sharing is not applicable to this article as no datasets will be generated or analyzed during the current study.

## Abbreviations

CADTH: Canadian Agency for Drugs and Technologies in Health
CT: Computed Tomography
DI: Diffusion imaging
DTI: Diffusion tensor imaging
DWI: Diffusion weighted imaging
fMRI: Functional magnetic resonance imaging
fNIRS: Functional Near-infrared Spectroscopy
MEG: Magnetoencephalography
MRI: Magnetic resonance imaging
PET: Positron Emission Tomography
PRISMA-ScR: Preferred Reporting Items for Systematic Reviews and Meta-Analyses extension for Scoping Reviews
SLP: Speech-language pathologist
SPECT: Single photon emission computed tomography
VBM: Voxel-based morphometry
