# Supplementary material for: Neuroimaging in pediatric language development and disorders: a scoping review protocol
Source: Syst Rev. 2025 Nov 21;14:235. doi: 10.1186/s13643-025-02969-y (PMC12639962; doi:10.1186/s13643-025-02969-y)
Supplement: Supplementary file 2 — Supplementary Material 2. Appendix 2: Search strategy for Medline [file 13643_2025_2969_MOESM2_ESM.docx]

Appendix 2 Search strategy

This proposed search strategy has been developed in consultation with a faculty liaison & instruction librarian of University of Toronto - St. George campus to ensure a sensitive search and that the key literature is captured. The initial search will be conducted in 6 databased: MEDLINE, Embase, EBSCO CINAHL, PsycINFO®, Scopus, and The Cochrane Library. The reference lists of identified articles will then be searched manually for additional studies.

An example of the search to be carried out in Medline is shown below.

**MEDLINE search**

The search strategy involves entering search terms using the Medline default [All Fields] setting within the Advanced Search function, having cleared all default filters. Using the [All Fields] setting in Medline draws upon Medical Subject headings (MeSH) without limiting to specific qualifiers or subheadings.

1. child/ or child, preschool/ or infant/ or infant, newborn/
2. (toddler* or preschool* or p#ediatric* or boy* or girl*). ti, ab, kw.
3. positron-emission tomography/ or positron emission tomography computed tomography/ or tomography, emission-computed, single-photon/ or single photon emission computed tomography computed tomography/ or diffusion tensor imaging/ or functional neuroimaging/ or perfusion imaging/ or spectroscopy, near-infrared/ or magnetic resonance imaging/ or diffusion magnetic resonance imaging/ or ultrasonography/ or magnetoencephalography/
4. (voxel-based morphometry or MR spectroscopy or Diffusion Weighted Imag* or Computed Tomography or ultrasound). ti,ab,kw.
5. (diffusion adj3(imaging or image or images)). ti,ab,kw.
6. language development/ or child language/
7. (language acquisition or language learn* or language process* or language function* or language abilit* or language network*).ti,ab,kw.
8. language disorders/ or agraphia/ or anomia/ or dyslexia/ or language development disorders/ or specific language disorder/
9. (language disorder* or language impairment* or language delay or delayed language or language disabilit* or developmental language disorders or specific language impairment*). ti,ab,kw.
10. (read* or writ* or syntax or semantic* or vocabulary or word* or gramma* or sentence* or literacy or story telling or narrat* or expressive or lexic*). ti,ab,kw.
11. 1 or 2
12. 3 or 4 or 5
13. 6 or 7 or 8 or 9 or 10
14. 11 and 12 and 13
