# Supplementary material for: Neuroimaging in pediatric language development and disorders: a scoping review protocol
Source: Syst Rev. 2025 Nov 21;14:235. doi: 10.1186/s13643-025-02969-y (PMC12639962; doi:10.1186/s13643-025-02969-y)
Supplement: Supplementary file 3 — Supplementary Material 3. Appendix 3: Title and abstract screening form [file 13643_2025_2969_MOESM3_ESM.docx]

Title and abstract screening form

Reviewer:

Inclusion criteria:

(1) children under eight years old;

(2) typically developing children or children with language disorders;

(3) studies involving neuroimaging techniques as research methodology;

(4) a primary focus on language development and/or language disorders

| Paper ID | Title | Decision  (Yes, No or Maybe) | Annotations  (Reason to exclude or be uncertain) |
| --- | --- | --- | --- |
|  |  |  |  |
|  |  |  |  |
|  |  |  |  |
|  |  |  |  |
|  |  |  |  |
|  |  |  |  |
|  |  |  |  |
|  |  |  |  |
|  |  |  |  |
|  |  |  |  |
|  |  |  |  |
|  |  |  |  |
|  |  |  |  |
|  |  |  |  |
|  |  |  |  |
|  |  |  |  |
|  |  |  |  |
|  |  |  |  |
|  |  |  |  |
|  |  |  |  |
|  |  |  |  |
|  |  |  |  |
|  |  |  |  |
|  |  |  |  |
|  |  |  |  |
|  |  |  |  |
|  |  |  |  |
|  |  |  |  |
