# Supplementary material for: Neuroimaging in pediatric language development and disorders: a scoping review protocol
Source: Syst Rev. 2025 Nov 21;14:235. doi: 10.1186/s13643-025-02969-y (PMC12639962; doi:10.1186/s13643-025-02969-y)
Supplement: Supplementary file 5 — Supplementary Material 5. Appendix 5: Data extraction form [file 13643_2025_2969_MOESM5_ESM.docx]

Data extraction form

Extractor:

RQs:

1. What neuroimaging techniques have been utilized to study language development and language disorders in pediatric populations?
2. How does neuroimaging contribute to understanding the neural mechanisms underlying language development and language disorders in children under 8 years old?
3. What are the challenges and limitations of using neuroimaging in pediatric language development and disorder research?

| Paper ID | Authors | Publication year | Publication type (Published/ unpublished) | Study type | Age group | Condition  (TD or with language disorders) | Neuroimaging techniques used | Primary focus | Outcome measures | Main findings |
| --- | --- | --- | --- | --- | --- | --- | --- | --- | --- | --- |
|  |  |  |  |  |  |  |  |  |  |  |
|  |  |  |  |  |  |  |  |  |  |  |
|  |  |  |  |  |  |  |  |  |  |  |
|  |  |  |  |  |  |  |  |  |  |  |
|  |  |  |  |  |  |  |  |  |  |  |
|  |  |  |  |  |  |  |  |  |  |  |
|  |  |  |  |  |  |  |  |  |  |  |
|  |  |  |  |  |  |  |  |  |  |  |
|  |  |  |  |  |  |  |  |  |  |  |
|  |  |  |  |  |  |  |  |  |  |  |
|  |  |  |  |  |  |  |  |  |  |  |
